# Transcriptome sequencing reveals iron acquisition–related genes and iron acquisition systems in *Auricularia cornea*

**DOI:** 10.1186/s12864-026-12654-6

**Published:** 2026-02-26

**Authors:** Qiushi Zhu, Ming Fang, Fangjie Yao, Lixin Lu, Xiaoxu Ma, Yuling Cui, Xu Sun, Zhen Wang

**Affiliations:** 1 Lab of the Genetic Breeding of Edible Mushroom, College of Horticulture, Jilin Agricultural University, Changchun, 130118 China; 2 Engineering Research Center of Edible and Medicinal Fungi, Ministry of Education, Jilin Agricultural University, Changchun, 130118 China

**Keywords:** Auricularia cornea, Iron acquisition system, Siderophore, Transcriptome, Iron content

## Abstract

*Auricularia cornea* exhibits significant potential for iron-enrichment. However, the mechanisms behind iron absorption and accumulation remain poorly understood. This study employed biochemical and transcriptomic approaches to systematically investigate the effects of an iron-rich environment on iron accumulation in *A. cornea* across 3 growth stages, as well as to characterize its iron acquisition–related (IAR) genes and iron acquisition systems. Our findings showed that *A. cornea* can produce siderophores. Supplementation with Fe₂(SO₄)₃ markedly increased iron content in both mycelia and primordia. Transcriptomic analysis revealed that iron supplementation significantly altered the expression of key IAR genes during the mycelial stage, but not in the primordia and fruiting body stages. Nine key IAR genes were identified, indicating that *A. cornea* possesses the molecular basis for the iron accumulation via reductive and non-reductive iron acquisition systems and ferrous transport systems. Phylogenetic analysis revealed that proteins involved in iron transport were highly conserved across *Basidiomycota* and *Ascomycota*, whereas those with redox functions have undergone divergence. These results provide new insights into the iron acquisition in *A. cornea*.

**Supplementary Information:**

The online version contains supplementary material available at 10.1186/s12864-026-12654-6.

## Introduction

*Auricularia cornea* is a widely cultivated edible fungus valued for its rich nutritional composition, including abundant polysaccharides, low-fat proteins, vitamins, and essential mineral elements such as iron and potassium [1]. The iron content in its dried fruiting body can reach up to 400 mg/kg [2], a level that surpasses that of most vegetables and legumes [3]. Owing to its straightforward cultivation requirements and adaptability to various substrates, *A. cornea* is not only the most widely distributed species within its genus but also one of the most extensively cultivated edible fungi globally [4].

Iron is an essential micronutrient for nearly all living organisms, playing a critical role in numerous physiological processes, including oxygen transport, DNA synthesis, and electron transfer through its involvement in oxidation–reduction reactions [5, 6]. It has also been implicated in developmental processes such as sex differentiation in mammalian embryos [7]. In humans, conventional iron supplements are typically absorbed as free ferrous ions, yet their bioavailability is often limited by multiple physiological factors. Furthermore, excess free Fe^2+^ can catalyze the formation of free radicals, resulting in cellular membrane damage and lipid peroxidation [8]. In contrast, edible fungi can accumulate minerals during growth and incorporate them into functional organic compounds [9], representing an excellent source of non-heme iron for human nutrition.

Iron is the fourth most abundant element in the Earth’s crust and one of the most common transition metals. Despite its prevalence, iron predominantly exists in forms with low bioavailability. As a result, organisms must expend considerable energy to acquire and solubilize iron into a form that is bioavailable [5]. To overcome this challenge, microorganisms have evolved diverse strategies to enhance iron solubility. Fungi, in particular, employ multiple iron acquisition systems that often operate simultaneously, including both reductive and non-reductive iron acquisition systems [10]. Nearly all fungi express a non-reductive iron acquisition system specific for siderophore–iron complexes [11]. These complexes are internalized in fungi through transporters belonging to the Aerobactin/Related siderophore transporter (ARN)/Siderophore Iron Transporter (SIT) subfamily of the major facilitator superfamily (MFS) [12]. Notably, siderophore transporters represent one of the 17 gene families unique to fungi, with no homologs identified in prokaryotes or other eukaryotes [11]. Research on siderophores dates back to the 1990s, with significant advances resulting from structural and regulatory gene characterizations in model fungi such as *Aspergillus nidulans* and *Neurospora crassa* [13]. Following cellular uptake of the Fe^3+^–siderophore complex, iron is released via enzymatic hydrolysis of the siderophore, which represents a characteristic feature of the non-reductive iron acquisition system [14].

Under conditions of low iron availability, most organisms acquire iron through the reductive iron acquisition system [15], which encompasses both high-affinity and low-affinity iron acquisition systems. Substrates for this system include not only siderophore–iron complexes but also Fe^3+^, iron chelates, and insoluble trivalent iron salts [16]. Notably, not all fungi employ this mechanism; for example, *A. nidulans* lacks a functional reductive iron acquisition system [17]. In the reductive iron acquisition system, environmentally persistent Fe^3+^ is first reduced to Fe^2+^ by ferric reductases. The resulting Fe^2+^ is then imported into the cell via a high-affinity transport complex composed of ferroxidase and permease. In this complex, it is reoxidized to Fe^3+^. This process defines the high-affinity iron acquisition system [18]. This system is homeostatically regulated: it is highly expressed under iron scarcity but strongly suppressed under iron-replete conditions [10]. The oxidation step is essential, as Fe^3⁺^ is not a substrate for the ferroxidase–permease complex [19]. When iron is readily available, the high-affinity iron acquisition system remains inactive, and iron uptake occurs primarily through low-affinity transporters [20]. Under hypoxic conditions, extracellular iron predominantly exists as Fe^2+^, enabling direct uptake via Fe^2+^ transporters without the need for surface ferric reductases—a system referred to as the ferrous transport system. Notably, the high-affinity iron acquisition system is nonfunctional under hypoxia [10]. Following the uptake of siderophore–Fe^3+^ complexes, iron reductases convert them into siderophore–Fe^2+^ complexes. Due to the low affinity of siderophores for Fe^2+^, this reduction promotes the dissociation of Fe^2+^ [14, 21, 22]. The released Fe^2+^ may then enter the cytoplasm through specific ferrous transporters if dissociation occurs extracellularly [21–23]. Alternatively, it can be reoxidized to Fe^3+^ and internalized via the ferroxidase–permease complex [21, 22].

*A. cornea* is an economically and nutritionally important edible fungus, recognized as a valuable dietary source of iron. Nevertheless, the molecular mechanisms underlying its iron acquisition remain poorly understood. To address this knowledge gap, we investigated iron content and siderophore production across the key developmental stages of *A. cornea* under controlled iron supplementation conditions. By integrating physiological assays with high-throughput RNA sequencing data, we identified the key iron acquisition–related (IAR) genes. This study lays the groundwork for developing a deeper molecular understanding of iron acquisition in *A. cornea*.

## Materials and methods

### Culture and sample collection of *A. cornea*

The strain ACP16 of *A. cornea* was provided by the Horticultural College of Jilin Agricultural University (Changchun, China). The substrate for the treatment group (T group) consisted of 78% sawdust, 20% wheat bran, 1% lime, and 1% gypsum, supplemented with Fe₂(SO₄)₃ at a concentration of 3571 mg/kg. The control group (CK group) substrate followed the same basal formulation without iron supplementation. Sterilized substrates from both groups were dispensed into 90 mm Petri dishes and overlaid with cellophane. Mycelia plugs of ACP16 were inoculated onto the cellophane and incubated at 25 °C in darkness. Mycelia were harvested once they reached the edge of the dish. For primordium and fruiting body induction, cultivation bags were prepared using the same substrate formulations as described above. Each bag contained 300 g of substrate with a moisture content of 55%–60%. 50 bags per group were sterilized at 121 °C for 2 h. Inoculation was performed using 3 biological replicates per group. After inoculation, the bags were incubated at 25 °C in darkness until full mycelial colonization was achieved, then transferred to a culture room maintained at 25 °C and 85%–90% relative humidity, under diffuse light conditions with adequate ventilation. Primordia were harvested at a diameter of 5–10 mm, and fruiting bodies were collected before spore formation. All samples, including mycelia, primordia, and fruiting bodies, were immediately frozen in liquid nitrogen and stored at -80 °C. Three biological replicates were collected for each developmental stage for subsequent RNA extraction and transcriptome sequencing.

### Determination of siderophore activity in *A. cornea*

The mycelia of strain ACP16 were cultured on Chrome Azurol S (CAS) detection medium (Qingdao Hope Bio-Technology Co., Ltd., Qingdao, China). The medium was prepared by dissolving 10.87 g of the powder in 1 L of deionized water, followed by sterilization at 116 °C for 30 min. Plates were poured aseptically under laminar flow conditions. After inoculation with ACP16 mycelia, the cultures were incubated under appropriate growth conditions. Siderophore production was confirmed by the presence of orange halos around the colonies, indicating a positive reaction [24].

### Determination of total iron content

The total iron content in the samples was quantified using inductively coupled plasma optical emission spectrometry (ICP-OES; iCAP RQ, Thermo Scientific, USA) following a previously established method [25]. Briefly, 0.3 g of each sample was accurately weighed into a polytetrafluoroethylene (PTFE) digestion vessel and digested using a microwave-assisted system. After cooling to 50 °C, the digestate was diluted to a constant volume with ultrapure water and analyzed by ICP-OES for iron quantification.

### RNA extraction, cDNA library construction, and illumina sequencing

Total RNA was extracted from all samples using TRIzol reagent. RNA concentration and purity were assessed using a NanoDrop 2000 spectrophotometer (Thermo Scientific, USA). Sequencing libraries were constructed with the NEBNext Ultra II RNA Library Prep Kit for Illumina (New England Biolabs Inc., Ipswich, Massachusetts, USA) starting from 1 µg of total RNA per sample. Library quality was evaluated using Agilent 2100 Bioanalyzer (Agilent Technologies Inc., USA). Finally, high-throughput sequencing was performed on the Illumina NovaSeq X Plus platform with a paired-end 150 bp (PE150) configuration.

### Bioinformatics analysis

Raw sequencing reads were processed to ensure data quality by removing adapter sequences, reads containing ambiguous bases (N), and low-quality reads where more than 50% of bases had a Phred score (Qphred) ≤ 20. The resulting clean reads were aligned to the reference genome (https://www.ncbi.nlm.nih.gov/assembly/GCA_002287115.1) using HISAT2 v2.1.0. Gene annotation was performed against multiple databases: KEGG [26, 27], KOG, Pfam, CAZy [28], NR [29], GO [30], Swiss-Prot [31], and TCDB [32]. Gene expression levels were quantified using HTSeq v0.9.1 and normalized by FPKM. Differential expression analysis was conducted with DESeq2 v1.38.3, with genes considered differentially expressed at a P-adjust ≤ 0.05 and |log₂FoldChange| ≥ 1. A union set of differentially expressed genes (DEGs) from all comparisons was clustered using the Pheatmap and ComplexHeatmap packages in R v4.2.3, based on Euclidean distance and complete linkage. Functional enrichment analysis of GO (Gene Ontology, https://geneontology.org/) and KEGG (Kyoto Encyclopedia of Genes and Genomes, https://www.kegg.jp/kegg/pathway.html) terms was performed using clusterProfiler v4.6.0, with a significance threshold of P-adjust ≤ 0.05 based on the hypergeometric test.

### RT-qPCR validation

To validate the RNA sequencing (RNA-seq) results, real-time quantitative polymerase chain reaction (RT-qPCR) was performed on selected DEGs. Gene-specific primers were designed using Primer Premier v6.0 (Additional Table S1) and synthesized by Kumei Biological Company (Changchun, China). Amplification reactions were carried out using the TransStart TOP Green RT-qPCR kit (TransGen Biotech, China), with EF1-a serving as the reference gene [33]. Relative gene expression levels were calculated using the 2^–ΔΔCt^ method [34]. All procedures, including reaction setup and thermal cycling, were performed in strict accordance with the manufacturer’s protocols.

### Protein modeling analysis and interaction prediction

Protein structure prediction was performed using AlphaFold3 v3.0.1 [35], with both predicted TM-score (pTM) and interface pTM (ipTM) values calculated for model evaluation. Protein–protein docking was carried out with HDOCKlite v1.1 [36]. The Spacing parameter was set to 1.2, and the Angle parameter was set to 15. Docking scores were converted into confidence scores using the following equation: Confidence_score = 1.0/[1.0 + e^0.02*(Docking_Score+150)^]. Active site analysis and structural visualization were conducted in PyMOL v3.10.

### Construction of the phylogenetic tree

To investigate the evolutionary relationships of key IAR proteins, the amino acid sequences encoded by the selected IAR genes (Additional Table S2) were used as queries to search for homologous sequences within the *Basidiomycota* and *Ascomycota* lineages in the NCBI non-redundant protein database using Proteins BLAST (BLASTP). Homologous sequences were retained based on the following criteria: query coverage ≥ 70%, sequence identity ≥ 35%, and E-value < 1 × 10⁻²⁰. When more than 50 homologous sequences were retrieved for a given query, the top 50 hits with the lowest E-values were selected for downstream analyses to ensure a manageable dataset size and representative coverage. Multiple sequence alignments of the retained high-homology sequences were performed using the ClustalW algorithm, as implemented in MEGA v12. Phylogenetic trees were subsequently constructed based on the aligned sequences using the Neighbor-Joining method. The robustness of tree topology was evaluated by bootstrap analysis with 1,000 replicates, and only well-supported clades were considered for evolutionary interpretation.

## Results

### Production of siderophore in *A. cornea* mycelia

**Fig. 1:**
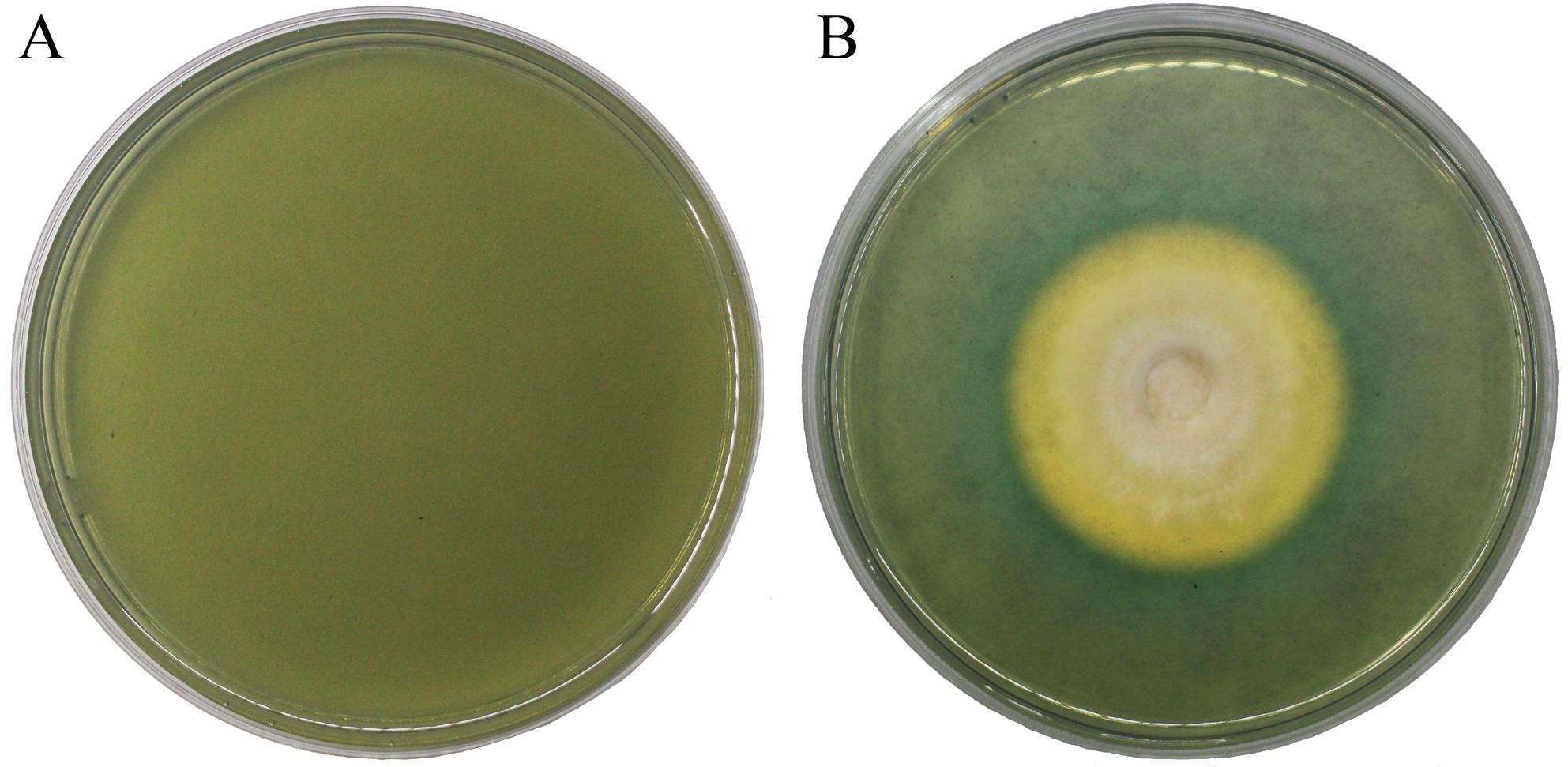
Growth of *A. cornea* mycelia on CAS medium. **A** Uninoculated CAS medium (negative control). **B** CAS medium inoculated with *A. cornea* mycelia

The results showed that the formation of a distinct orange-yellow halo surrounding the test strains’ colony was observed on blue agar medium. It indicated the ability of its hyphae to synthesize and secrete siderophores (Fig. 1).

### Total iron content in *A. cornea* at different growth stages

**Fig. 2:**
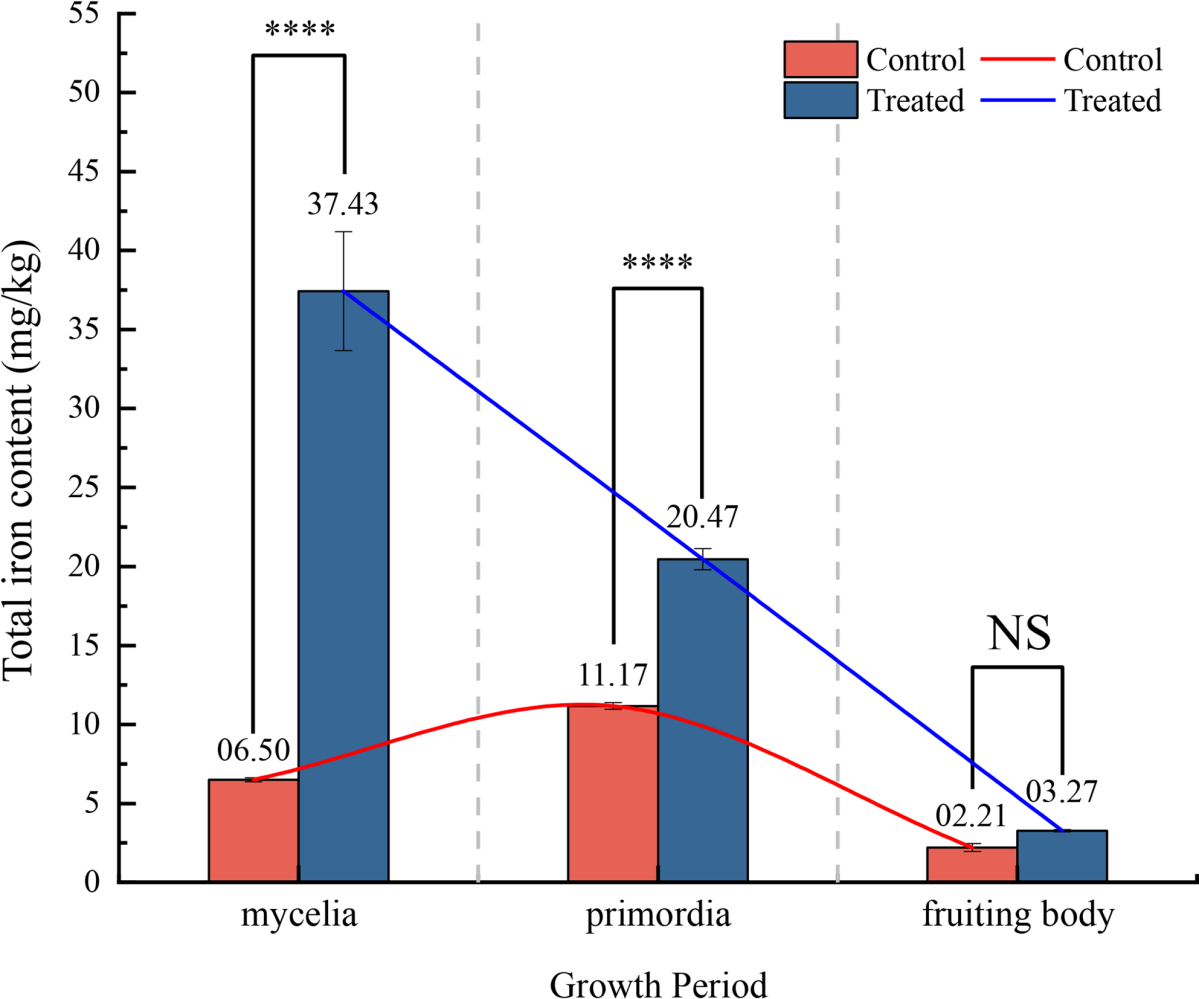
Changes in total iron content in *A. cornea* during 3 growth periods. Total iron content in *A. cornea* in 3 growth periods. Red bars and lines represent the CK group, blue bars and lines represent the T group. NS (*P* > 0.05), * (*P* ≤ 0.05), ** (*P* ≤ 0.01), *** (*P* ≤ 0.001), **** (*P* ≤ 0.0001). Error bars represent the standard deviation of total iron content measured by ICP-OES from 3 biological replicates

The total iron content in the mycelium stage of the T group was 37.43 mg/kg, compared with 6.50 mg/kg in the CK group, corresponding to an approximately 5.8-fold increase. In the primordia stage, the total iron content reached 20.46 mg/kg in the T group, whereas the CK group showed 11.16 mg/kg, representing an approximately 1.8-fold increase. In the fruiting body stage, the total iron content was 3.27 mg/kg for the T group and 2.21 mg/kg for the CK group. No significant difference was observed between the 2 groups at this stage. The total iron content in the T group exhibited a decreasing trend across the three growth stages. In contrast, the control group showed an initial increase followed by a decrease (Fig. 2).

### Reliability assessment of RNA sequencing datasets

To assess data reliability, correlation analysis was performed across all transcriptome samples, revealing strong consistency among the 3 biological replicates within each group (Additional Fig S1A). Principal component analysis (PCA) further demonstrated clear separation of transcriptomic profiles according to developmental stage (mycelia, primordia, and fruiting body) (Additional Fig S1B). To validate the RNA-seq results, 6 DEGs were randomly selected for RT-qPCR analysis. Their expression patterns showed strong agreement with the transcriptomic data (Additional Fig S2).

### Differentially-expressed genes

**Fig. 3:**
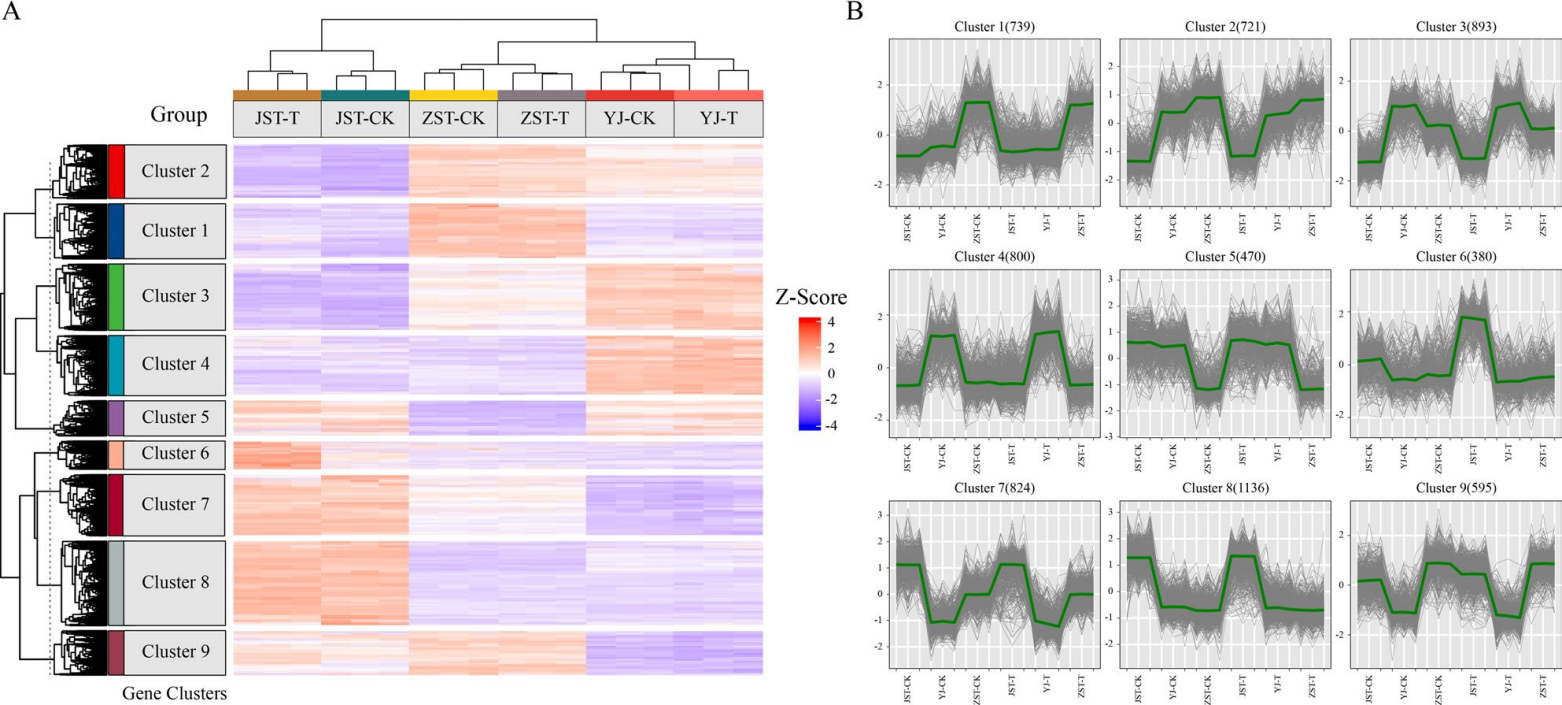
Gene Clusters and Z-score Expression Profiles of Samples in Different Groups. **A** Heatmap of clustering analysis on gene expression pattern comparison. **B** Cluster analysis of gene expression trends. The background line in the figure shows the expression pattern of genes in each cluster, and the middle line represents the average expression level of all genes in the cluster in the sample. Note: JST, YJ, and ZST represent the samples of mycelia, primordia, and fruiting bodies, T represents the treatment group and CK represents the control group

Comparative analysis between the CK and T groups identified 625 (Upregulated: 343, Downregulated: 282), 177 (Upregulated: 63, Downregulated: 114), and 161 (Upregulated: 54, Downregulated: 107) DEGs at the mycelial (JST-CK vs. JST-T), primordia (YJ-CK vs. YJ-T), and fruiting body (ZST-CK vs. ZST-T) stages, respectively, based on the thresholds of |Log₂(FoldChange)| ≥ 1 and adjusted p-value (padj) ≤ 0.05. Within the T group, 4095 (Upregulated: 1936, Downregulated: 2159) and 2852 (Upregulated: 1393, Downregulated: 1459) DEGs were detected during the transitions from mycelium to primordia (JST-T vs. YJ-T) and from primordia to fruiting body (YJ-T vs. ZST-T), respectively. Similarly, the CK group exhibited 3873 (Upregulated: 1890, Downregulated: 2073) and 2419 (Upregulated: 1229, Downregulated: 1262) DEGs in the corresponding developmental transitions (Table 1). Z-score-normalized expression values across all samples revealed distinct transcriptional profiles between the T and CK groups (Fig. 3A). Cluster analysis classified all DEGs into 9 expression clusters (Fig. 3B). Pronounced differences in expression patterns between the 2 groups were primarily observed at the mycelial stage, particularly in clusters 6 and 9. In contrast, gene expression profiles were largely similar between groups in the primordia and fruiting body stages.

**Table 1:** The DEGs in *A. cornea* under rich iron environment (|log_2_(FoldChange)|≥1 and padj ≤ 0.05)

Species/treatments	Nodiff	Upregulated	Downregulated
JST-CK vs. JST-T	11,262	343	282
YJ-CK vs. YJ-T	11,808	63	114
ZST-CK vs. ZST-T	11,843	54	107
JST-CK vs. YJ-CK	7970	1890	2073
YJ-CK vs. ZST-CK	9549	1229	1262
JST-T vs. YJ-T	8057	1936	2159
YJ-T vs. ZST-T	9240	1393	1459

### GO and KEGG enrichment analyses of DEGs

The DEGs of the T group vs. the CK group were annotated with GO enrichment analysis (Additional Fig S3). The DEGs were categorized into biological processes (BP), cellular components (CC), and molecular functions (MF). The results showed that several key terms were significantly enriched. In the BP category, DEGs were predominantly associated with “metal ion transport”, “organic hydroxy compound biosynthetic process”, “secondary metabolite biosynthetic process”, and “secondary metabolic process”, suggesting that iron supplementation influences cellular processes related to metal ion transport and secondary metabolism. Within the CC category, significant enrichment was observed for “ER membrane insertion complex” and “TRC complex”, indicating that iron availability affects the synthesis and localization of membrane-associated proteins. In the MF category, a significant number of DEGs were enriched in “oxidoreductase activity”, implying that iron regulates the expression of genes involved in redox reactions.

KEGG pathway enrichment analysis of all DEGs revealed three significantly enriched metabolic pathways in the mycelia of the T group compared to the CK group: Nitrogen metabolism (map00910), Other glycan degradation (map00511), and Fatty acid degradation (map00071) (Additional Fig S4). In contrast, no pathways were significantly enriched among DEGs at either the primordia or fruiting body stages.

KEGG pathway enrichment analysis of DEGs from the mycelia to primordia transition (YJ vs. ZST) was performed. Ten significantly enriched pathways were identified in the T group (Additional Fig S5B), while only 1 was identified in the CK group (Additional Fig S5A). Comparative analysis revealed 9 pathways unique to the T group (Additional Fig S5), which were functionally categorized as follows: 5 in carbohydrate metabolism, 2 in amino acid metabolism, 1 in lipid metabolism, and 1 in energy metabolism. Functional interpretation suggested these pathways contribute to critical developmental processes, including cell wall biosynthesis, membrane formation, polysaccharide synthesis, the tricarboxylic acid (TCA) cycle, gluconeogenesis, abiotic stress response, and energy supply under hypoxic conditions.

KEGG pathway enrichment analysis of DEGs from the primordia to fruiting body transition (YJ vs. ZST) was conducted. Ten enriched pathways were identified in the T group (Additional Fig S6A), while 8 were identified in the CK group (Additional Fig S5B). Comparative analysis revealed 4 pathways unique to the T group (Additional Fig S5), comprising 2 related to carbohydrate metabolism, 1 to lipid metabolism, and 1 involved in the metabolism of cofactors and vitamins. Functional analysis suggested these pathways are implicated in cell wall biosynthesis, gluconeogenesis, and abiotic stress response. The gene IDs of stage-specific DEGs between the CK and T groups, along with their GO terms, KEGG annotations, and KEGG pathways, are provided in Additional Table S6.

### Identification of candidate IAR genes and selection of key genes

**Fig. 4:**
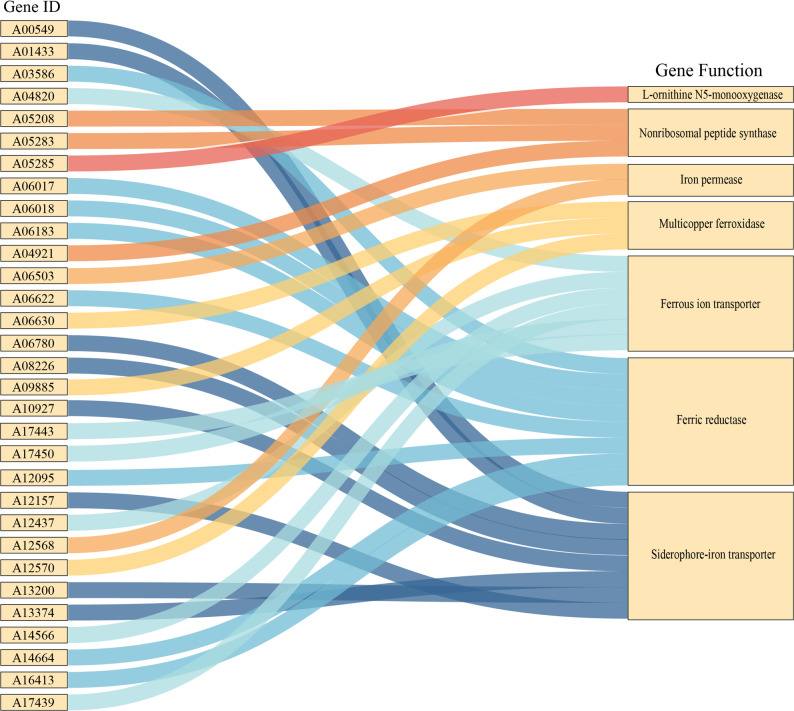
IAR gene IDs and functions in *A. cornea*

**Fig. 5:**
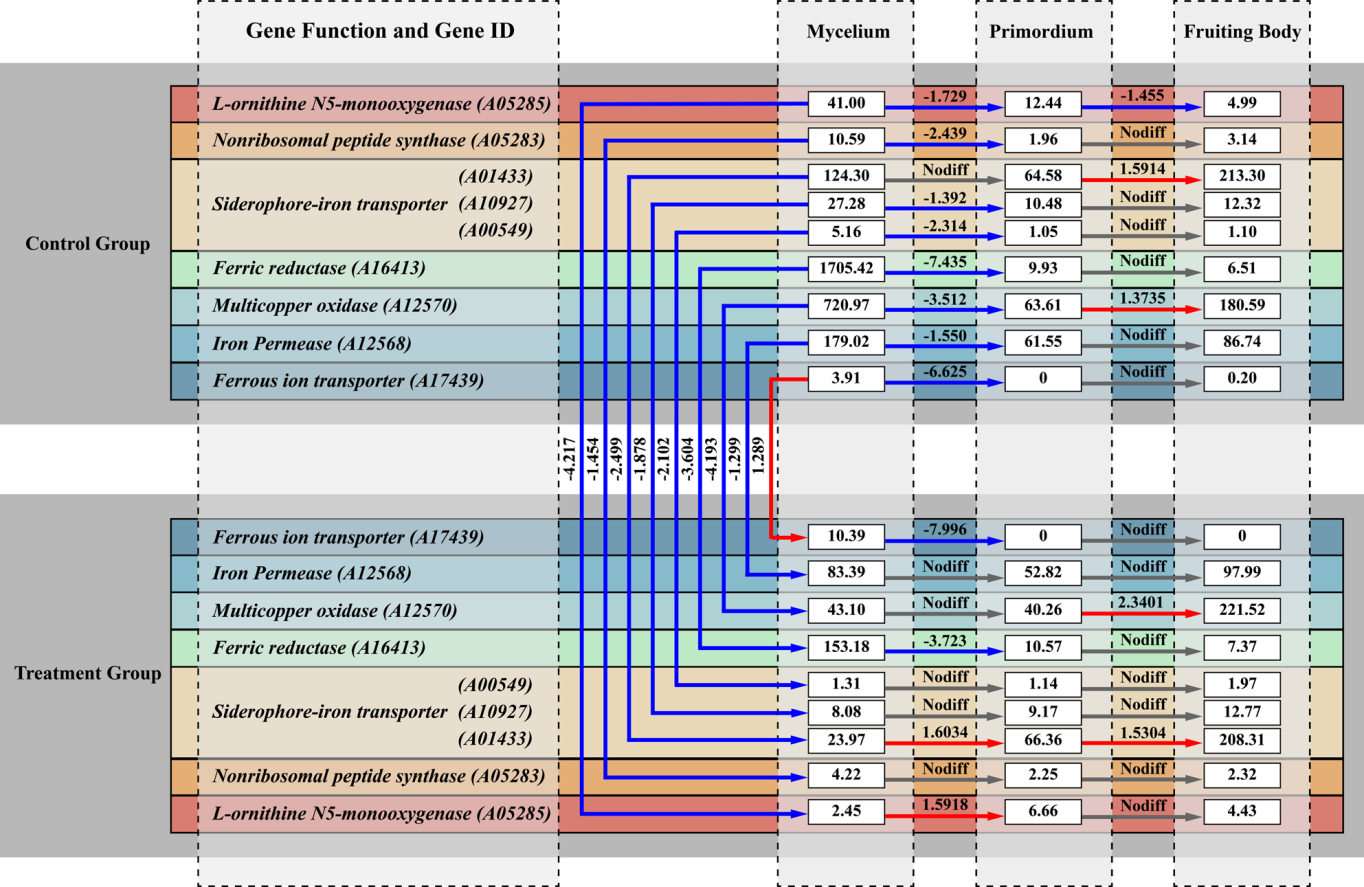
FPKM and Log_2_(FoldChange) values for key IAR genes across 3 growth stages. Blue lines indicate downregulated genes, red lines indicate upregulated genes, and grey lines indicate no significant difference. Values along the lines represent Log_2_(FoldChange) between the T group and CK group, while values within boxes indicate FPKM values for each gene

Based on functional annotation using the KEGG [26, 27], KOG, Pfam, CAZy [28], NR [29], GO [30], Swiss-Prot [31], and TCDB [32]databases, a total of 31 IAR genes were identified (Fig. 4, Additional Table S3). Based on differential expression analysis between the T and CK groups at the mycelial stage, nine key IAR genes were subsequently screened. Eight of these genes were downregulated, including 1 *Nonribosomal peptide synthase*, 1 *L-ornithine N⁵-monooxygenase*, 3 *Siderophore-iron transporters*, 1 *Ferric reductase*, 1 *Multicopper ferroxidase*, and 1 *Iron permease*. Concurrently, 1 *Ferrous ion transporter* was upregulated (Fig. 5, Additional Table S4). No IAR DEGs were detected at either the primordia or fruiting body stages. Among them, nonribosomal peptide synthases and L-ornithine N⁵-monooxygenases are involved in the biosynthesis of siderophores; siderophore-iron transporters mediate the transport of siderophore–iron complexes across membranes; ferric reductases catalyze the reduction of Fe^3+^ to Fe^2+^; multicopper ferroxidases and iron permeases can assemble into a membrane ferroxidase–permease complex. In this system, the ferroxidase oxidizes Fe^2+^ to Fe^3+^, and the permease subsequently mediates the uptake of Fe^3+^ into the cell; in addition, ferrous ion transporters are responsible for the transmembrane transport of Fe^2+^.

Comparative analysis of IAR genes across developmental stages under identical treatments revealed distinct expression dynamics. In the T group, comparative analysis of key IAR genes from mycelia to primordia (JST vs. YJ) revealed that 2 genes were downregulated: *Ferric reductase* (*A16413*) and *Ferrous ion transporter* (*A17439*). Meanwhile, 2 other genes were upregulated: *L-ornithine N⁵-monooxygenase* (*A05285*) and *Siderophore-iron transporter* (*A01433*). The remaining 5 genes showed no significant expression change. In the subsequent transition from primordia to fruiting body (YJ vs. ZST), 2 genes were upregulated: *Siderophore-iron transporter* (*A01433*) and *Multicopper oxidase* (*A12570*). The remaining 7 genes showed no significant expression change (Fig. 5, Additional Table S4).

In the CK group, comparative analysis of key IAR genes from mycelia to primordia (JST vs. YJ) revealed that 8 genes were downregulated, while the *Siderophore-iron transporter* (*A01433*) showed no significant change in expression. During the subsequent transition from primordia to fruiting body (YJ vs. ZST), 2 genes were upregulated: *Siderophore-iron transporter* (*A01433*) and *Multicopper oxidase* (*A12570*); 1 gene was downregulated: *L-ornithine N⁵-monooxygenase* (*A05285*); and the remaining 6 genes exhibited no significant differential expression (Fig. 5, Additional Table S4).

### Molecular biology prediction of interacting proteins

**Fig. 6:**
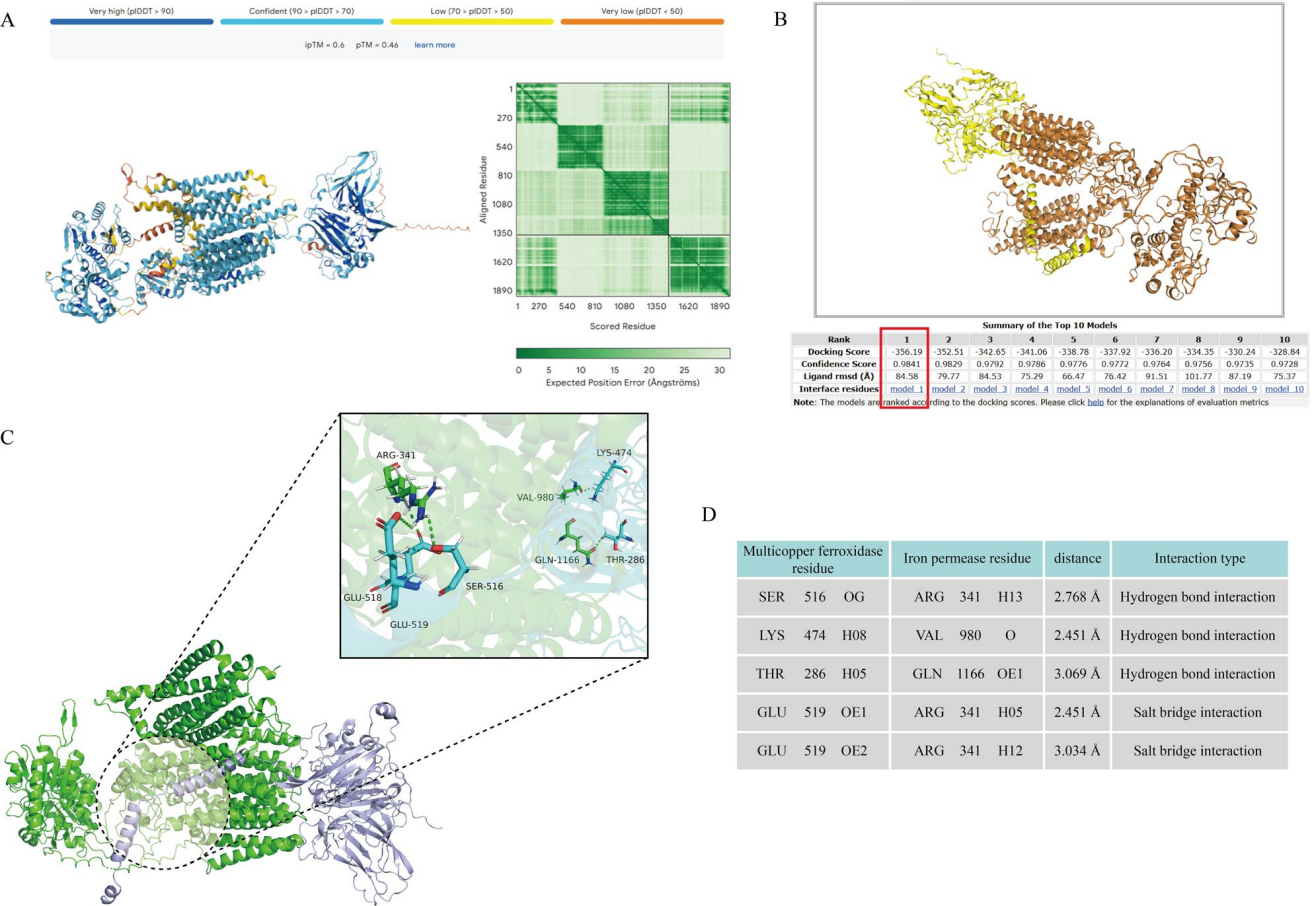
Computational simulation of the interaction mechanism between multicopper ferroxidase and Iron Permease and characterization of the binding site. **A** AlphaFold3 conformational prediction analysis results of Multicopper ferroxidase and Iron permease. **B** HDOCK protein–protein docking analysis results of Multicopper ferroxidase and Iron permease. **C** Core site mapping. Interacting amino acids are shown as stick models; the Iron permease protein molecule is represented by a green helical structure, and the Multicopper ferroxidase protein molecule is represented by a purple helical structure. **D** Core site information

The three-dimensional structures of Multicopper ferroxidase (Gene ID: *A12570*) and Iron permease (Gene ID: *A12568*) were predicted using AlphaFold v3.0.1, and their interaction was analyzed by protein-protein docking with HDOCKlite v1.1. Core binding sites were mapped using PyMOL v3.10 (Fig. 6A-C). The resulting model showed high reliability, with a pTM+ipTM score of 1.06 (threshold > 0.75), a Confidence Score of 0.9841 (threshold > 0.7), and a Docking Score of -356.1 (threshold <-200). Five core interaction sites were identified between the 2 proteins, including 3 hydrogen bond interactions and 2 salt bridge interactions (Fig. 6D). These results collectively indicate that the predicted protein complex exhibits structural stability and biological plausibility, supporting its potential functional relevance in iron acquisition.

### Phylogenetic relationships of key genes involved in iron acquisition

Phylogenetic analysis based on amino acid sequence alignments revealed that siderophore–iron transporters (A10927, A00549), iron permease (A12568), and ferrous ion transporter (A17439) exhibited a high degree of sequence conservation across both *Basidiomycota* and *Ascomycota*. In contrast, L-ornithine N⁵-monooxygenase (A05285) and nonribosomal peptide synthase (A05283) showed clear phylogenetic separation between the *Basidiomycota* and *Ascomycota* lineages. In addition, among the homologous amino acid sequences retrieved, no highly homologous sequences corresponding to ferric reductase (A16413), multicopper oxidase (A12570), or siderophore–iron transporter (A01433) were identified in *Ascomycota* (Additional Fig S7).

## Discussion

### Iron acquisition system in *A. cornea* and evolution of related genes

**Fig. 7:**
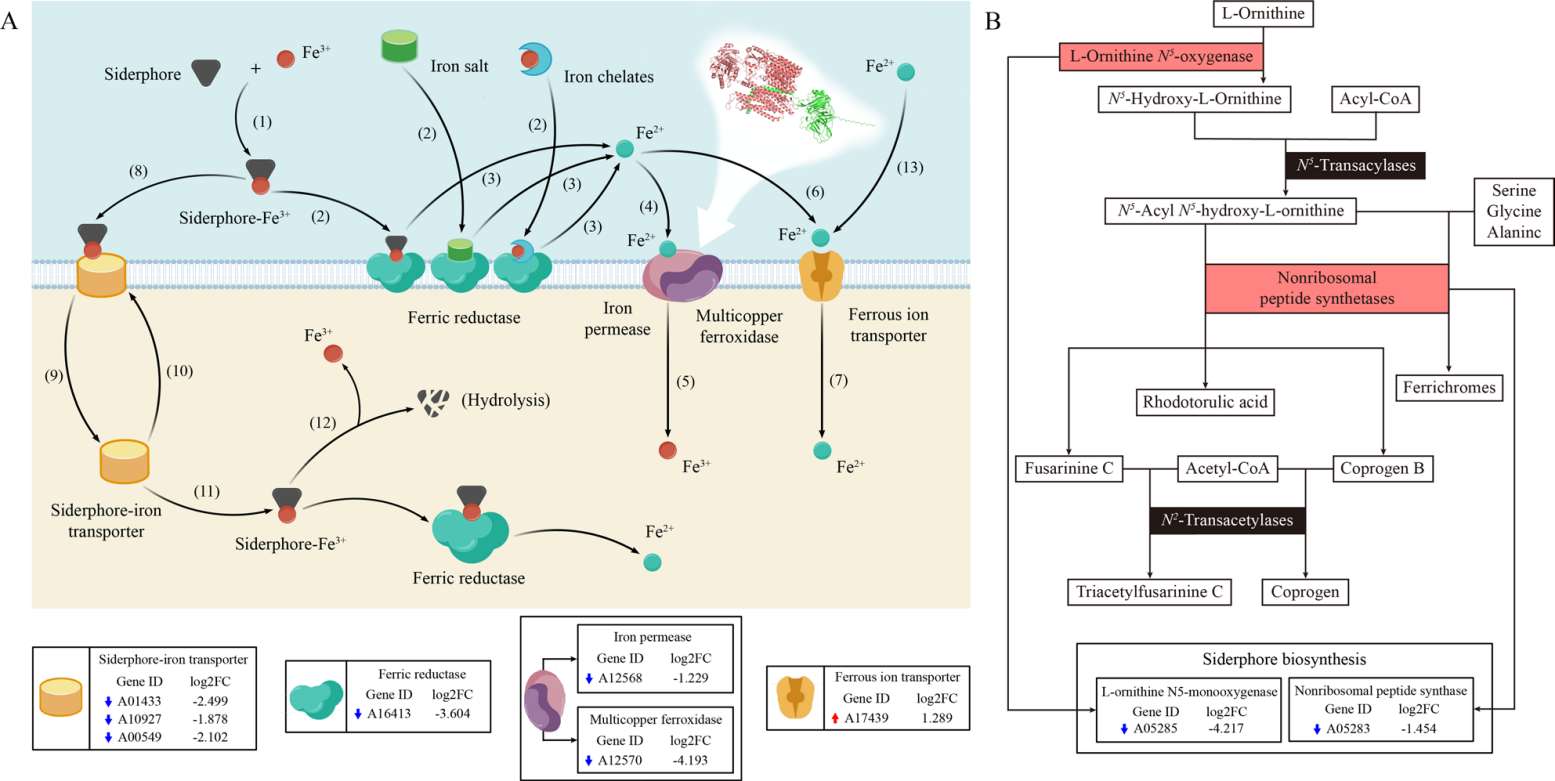
Iron acquisition systems and biosynthesis of siderophores in *A. cornea*. **A** Iron acquisition systems [10, 38]. (1), (2), (3), (4), (5): high-affinity iron acquisition systems; (1), (2), (3), (6), (7): low-affinity iron acquisition systems; (1), (8), (9), (10), (11), (12): non-reductive system; (13), (7): ferrous transport system. **B** Biosynthesis of siderophores [39–41]. The blue downward arrow represents the downregulation of expression in the T group relative to the CK group, and the red upward arrow represents the upregulation of expression in the T group relative to the CK group

Nine key IAR genes in *A. cornea* were, for the first time, identified in this study. Among them, *L-ornithine N⁵-monooxygenase* and *Nonribosomal peptide synthase* are key genes for siderophore biosynthesis; *Siderophore-iron transporter* is a key gene for the non-reductive iron acquisition system; *Ferric reductase*, *Multicopper oxidase*, and *Iron permease* are key genes for the high-affinity iron acquisition system; *Ferric reductase* and *Ferrous ion transporter* are key genes for the low-affinity iron acquisition system. Collectively, these results indicate that *A. cornea* synthesizes siderophores through the Non-Ribosomal Peptide Synthetase (NRPS) pathway and employs multiple iron uptake strategies, including the reductive iron acquisition system, non-reductive iron acquisition system, and ferrous transport system (Fig. 7). In contrast, fungi such as *Saccharomyces cerevisiae*, *Candida albicans*, and *Cryptococcus neoformans* lack autonomous siderophore synthesis and rely on the uptake of xenosiderophores via the non-reductive iron acquisition system [10, 37]. Similarly, *A. nidulans*, which lacks a functional reductive iron acquisition system, depends exclusively on the non-reductive iron acquisition system and ferrous transport system for iron acquisition [17]. The presence of genetic components supporting 3 distinct iron acquisition systems in *A. cornea* suggests a robust and versatile capacity for iron uptake, which may contribute to its adaptability across varying environmental conditions.

Nine key IAR genes in *A. cornea* were, for the first time, identified in this study. The transport-related proteins encoded by these genes exhibited relatively high conservation between *Basidiomycota* and *Ascomycota*. In contrast, redox-functional proteins underwent significant divergence during the evolution of these 2 fungal phyla. Notably, proteins involved in siderophore biosynthesis, including L-ornithine N⁵-monooxygenase and nonribosomal peptide synthase, displayed marked phylogenetic divergence. Overall, the IAR proteins in *A. cornea* have undergone distinct evolutionary trajectories, characterized by varying degrees of sequence conservation and divergence. Given the exploratory nature of this study, the phylogenetic results were primarily used to identify broad patterns of conservation and divergence, rather than to infer detailed evolutionary histories.

### Analysis of the downregulation of key IAR genes in mycelia and the consequent increase in iron content under high-iron conditions

Transcriptome analysis showed that exogenous supplementation with 3571 mg/kg Fe₂(SO₄)₃ resulted in the downregulation of 8 out of 9 key IAR genes at the mycelial stage. Based on the observed expression patterns and iron content measurements, this transcriptional response is inferred to involve at least 2 potential regulatory mechanisms. First, the repression of IAR genes under iron-replete conditions is widely recognized as a conserved feedback strategy in fungi to maintain iron homeostasis and prevent iron-mediated toxicity. Existing studies have demonstrated that both Fe^2+^-mediated Fenton reactions and Fe^3+^-catalyzed Haber–Weiss reactions can generate hydroxyl radicals (OH•) [42], which subsequently induce peroxidation of polyunsaturated fatty acids in the cell membrane, disrupt the stability of the lipid bilayer, and ultimately lead to cellular damage [43]. Consequently, organisms have evolved sophisticated mechanisms to regulate iron acquisition, utilization, and storage [5, 39]. In the present study, exogenous iron supplementation resulted in the downregulation of 8 out of 9 key IAR genes during the mycelial stage, including those involved in siderophore biosynthesis, the high-affinity iron acquisition system, and the non-reductive iron acquisition pathway. Similar iron-responsive regulatory strategies have been widely reported in diverse fungal species. In many *Ascomycota* and *C. neoformans* (*Basidiomycota*), iron homeostasis is transcriptionally regulated by GATA-family transcription factors. Under iron-replete conditions, these transcription factors directly repress the expression of IAR genes as well as genes encoding regulatory components of the CCAAT-binding complex [44]. In *Aspergillus fumigatus*, the transcription factor HapX activates vacuolar iron storage under iron-replete conditions, thereby enhancing fungal tolerance to high-iron environments [45, 46]. Second, oxygen availability may also contribute to the observed transcriptional patterns. In this study, iron supplementation was accompanied by downregulation of most IAR genes at the mycelial stage, consistent with previous reports [10, 47, 48], while the *ferrous iron transporter* was concurrently upregulated. Under hypoxic or low-oxygen conditions, the oxidation of Fe^2+^ to Fe^3+^ becomes less efficient, favoring direct Fe^2+^ uptake via ferrous transport systems [10]. Notably, differentially expressed genes between the mycelial and primordia stages in the iron-supplemented group were significantly enriched in the methane metabolism pathway. Although methane metabolism enrichment cannot be considered direct evidence of hypoxia, intermediates such as formic acid may serve as alternative energy sources under oxygen-limited or reducing conditions, providing indirect support for altered oxygen availability within the cultivation system. Despite the transcriptional repression of key iron uptake genes, mycelial iron content increased significantly under high-iron conditions. The sampling time point may partly explain this apparent discrepancy. Mycelia were collected 15 days post-inoculation, when the cultures had fully colonized the medium, and iron accumulation may have already reached a relatively high and stable level. At this stage, further iron uptake may no longer be required, and IAR gene expression could be suppressed through iron-responsive negative feedback to minimize potential cytotoxic effects associated with excess intracellular iron. Consistent with this interpretation, previous studies have shown that fungal iron-responsive transcriptional regulation can occur rapidly following changes in iron availability; for example, HapX expression in *A. fumigatus* can be repressed within 30 min after a shift from iron-depleted to iron-replete conditions [47, 48]. Taken together, these interpretations are primarily inferred from transcriptomic analyses and iron content measurements and should be regarded as working hypotheses rather than definitive mechanisms. Future studies will require direct evidence, such as measurements of oxygen levels in the cultivation substrate, reactive oxygen species (ROS) levels, iron speciation, and subcellular iron localization, together with the collection of mycelial samples at multiple time points to assess iron content and transcriptomic profiles, to validate the regulatory pathways underlying IAR gene repression under iron-replete conditions and to elucidate the dynamic relationship between iron availability and gene expression during mycelial development.

### Iron acquisition strategy for *A. cornea*

The total iron content in the T group exhibited a progressive decline across the 3 developmental stages, whereas the CK group showed an initial increase followed by a decrease. At both the mycelia and primordia stages, iron content was significantly higher in the T group than in the CK group; however, no significant intergroup difference was observed at the fruiting body stage. These results indicate that elevated iron concentration in the substrate significantly enhanced iron accumulation in the mycelia and primordia, but did not increase iron content in the fruiting body.

Transcriptomic analysis showed that during the developmental transition from mycelia to fruiting bodies, *siderophore–iron transporter* and *multicopper oxidase* were upregulated, whereas most other key IAR genes were markedly downregulated. Notably, the *ferrous ion transporter* was not detectably expressed at either the primordium or fruiting body stage in both the CK and T groups.

Based on the expression patterns, iron uptake in *A. cornea* is inferred to occur primarily during the mycelium stage. Meanwhile, during the later stages of fruiting body development, *A. cornea* may undergo a shift in its iron acquisition strategy. Specifically, the low-affinity iron acquisition system and the ferrous transport system are gradually downregulated. In contrast, *A. cornea* increasingly relies on the non-reductive iron acquisition system. In *Basidiomycota*, siderophore–iron complexes have been reported to function as an intracellular iron storage form [49]. Through the non-reductive iron acquisition system, iron is internalized in the form of siderophore–iron complexes, thereby reducing the accumulation of free Fe^2+^ and mitigating oxidative damage induced by Fenton reactions [50, 51]. This mechanism is therefore proposed to facilitate iron storage while potentially alleviating oxidative stress. Meanwhile, during the transition from mycelium to primordia and fruiting bodies, the overall capacity for iron acquisition may decrease due to the downregulation of most key IAR genes. Consequently, the combined effects of reduced iron uptake and increased biomass during the primordia and fruiting-body stages may lead to a decline in iron concentration. This likely explains the continuous decrease in iron content in the T group during the mycelium-to-fruiting-body transition, as well as the absence of a statistically significant difference in iron content between the T and CK groups at the fruiting-body stage.

### Synthesis of *A. cornea* siderophore

Previous studies have not documented siderophore production in *Auricularia* species [52]. However, recent genomic analyses have revealed that *Auricularia* species harbor a substantial number of NRPS and NRPS-like genes, highlighting their potential to synthesize diverse nonribosomal peptides [53]. Nevertheless, the specific involvement of these NRPSs in siderophore biosynthesis has not yet been experimentally validated.

In the present study, siderophore production was experimentally examined using the CAS assay. Mycelia from 6 strains (3 cultivated strains and 3 Wild strains) were cultured on potato dextrose agar (PDA), and 4 strains (2 cultivated strains and 2 Wild strains) were cultured on sawdust-based substrate. All prepared mycelia were then inoculated onto solid CAS detection medium (Additional Table S5). All tested strains formed distinct orange-yellow halos around the colonies against the blue agar background, confirming that *A. cornea* mycelia are capable of synthesizing and secreting siderophores.

Compared to the CK group, the DEGs identified in the mycelia stage in the T group included 2 key genes involved in the NRPS-dependent siderophore biosynthesis pathway: *L-ornithine N⁵-monooxygenase* (*A05285*) and *Nonribosomal peptide synthase* (*A05283*). Functional enrichment analysis revealed that these genes were significantly associated with the hydroxamate-containing siderophore biosynthetic process (GO:0019539), indicating that *A. cornea* synthesizes hydroxamate-type siderophores through the NRPS pathway. Based on these findings, 16 candidate siderophores were selected from the 918 structurally unique siderophores in the Siderophore Information Database SIDERTIE [54] using the following screening criteria: kingdom: Fungi, phylum: *Basidiomycota*, biosynthetic type: NRPS, and functional group: hydroxamate (Additional Fig S8). These candidates provide a reference set for future identification of siderophores in *A. cornea*.

## Conclusion

Research findings demonstrate that *A. cornea* is capable of siderophore production. Iron supplementation significantly enhanced iron accumulation in the mycelia and primordia, though it did not markedly affect iron content in the fruiting body. A general decline in iron content was observed throughout fungal development. Nine key IAR genes were identified, among which exogenous iron supplementation suppressed the expression of most genes at the mycelia stage, whereas no significant transcriptional changes were detected during the primordia and fruiting body stages. Phylogenetic analysis revealed that proteins involved in iron transport are highly conserved across *Basidiomycota* and *Ascomycota*, whereas those with redox functions have undergone divergence.

## Supplementary Information

Supplementary Material 1.

Supplementary Material 2.

Supplementary Material 3.

Supplementary Material 4.

Supplementary Material 5.

Supplementary Material 6.

Supplementary Material 7.

Supplementary Material 8.

Supplementary Material 9.

Supplementary Material 10.

Supplementary Material 11.

Supplementary Material 12.

Supplementary Material 13.

Supplementary Material 14.

## Data Availability

The datasets generated and analyzed during the current study are available in the NCBI Sequence Read Archive (SRA) repository, under project number PRJNA1368693. The website is: https://www.ncbi.nlm.nih.gov/bioproject/PRJNA1368693.

## Abbreviations

CAS: Chrome Azurol S
DEG: Differentially expressed gene
IAR: Iron acquisition-related
JST: Mycelia
YJ: Primordia
ZST: Fruiting body
ARN: Aerobactin/Related siderophore transporter
SIT: Siderophore Iron Transporter
MFS: Major facilitator superfamily
T group: Treatment group
CK group: Control group
ICP-OES: Inductively coupled plasma optical emission spectrometry
PTFE: Polytetrafluoroethylene
RT-Qpcr: Real-time quantitative polymerase chain reaction
pTM: Predicted TM-score
ipTM: Interface pTM
BLASTP: Proteins BLAST
RNA-seq: RNA sequencing
BP: Biological processes
CC: Cellular components
MF: Molecular functions
TCA: Tricarboxylic acid
NRPS: Non-ribosomal peptide synthetase
OH•: Hydroxyl radicals
ROS: Reactive oxygen species
PDA: Potato dextrose agar
