# Supplementary material for: Transcriptome sequencing reveals iron acquisition–related genes and iron acquisition systems in Auricularia cornea
Source: BMC Genomics. 2026 Feb 26;27:336. doi: 10.1186/s12864-026-12654-6 (PMC13041173; doi:10.1186/s12864-026-12654-6)
Supplement: Supplementary file 1 — Supplementary Material 1. [file 12864_2026_12654_MOESM1_ESM.docx]

Table S1. Primer of RT-qPCR

| Primer number | Sense Primer | TM(℃) | Anti-sense Primer | TM(℃) |
| --- | --- | --- | --- | --- |
| *A02043* | GCCGCCGTTGTGGATGTCAT | 61 | CGATGCCGAGCAGGTTGATGAA | 59 |
| *A01433* | GCACCACGACCTACCTGTACCA | 60 | GCACGAACCACGCGCAGATAA | 60 |
| *A08949* | TGACGCCTTCCGAATCCTCCAT | 62 | AAGCCCTCAAGCCCTCGATCTC | 62 |
| *A01880* | GCTTGAGGTCAACGAGGCAGTC | 60 | GACGAAGAAGGACGCGCACAA | 62 |
| *A10277* | AGGCGTGGCTCGGTATGTG | 59 | TTGTCGAAGCAGTCAGAGGGAT | 61 |
| *A03326* | ATGCCAGTTGTTTGACCTTGTG | 61 | ATGTTGACCTCGCCCGTGT | 61 |
| *EF1-a* | ATCGTCGCCGTCAACAAGATGG | 60 | AGCCTTCGTCTCCTTCGTCCAA | 60 |
