# Supplementary material for: Transcriptome sequencing reveals iron acquisition–related genes and iron acquisition systems in Auricularia cornea
Source: BMC Genomics. 2026 Feb 26;27:336. doi: 10.1186/s12864-026-12654-6 (PMC13041173; doi:10.1186/s12864-026-12654-6)
Supplement: Supplementary file 2 — Supplementary Material 2. [file 12864_2026_12654_MOESM2_ESM.docx]

Table S2 Gene ID and amino acid sequence

| Gene ID | Amino Acid Sequence |
| --- | --- |
| *A05285* | MSVEVPPPSPSPALLQTPPDQDLPIEHDNEPYDLIGVGFGPTHIALAVAF  AEERPGKRALFIDARETFAWHPALLLPASRMQISFLKDLATFRNPQSSFS  FVSYLHSFGVDRLAAFSNLGSWTPSRTEWASYLAWAAKRLENNVSWSSKV  VSVQSVPATDADTSLVKVTIHDVANQKMRVVYARNLSLAPGATPFIPGLF  AEALPAFPEIAHTSAYLSTLERLKLDPATFNGRVSVVGGGQSSAEVFHDL  ISRFPHAKVDMFYRASALVPADDSPFVNARAFDPARTDEFFSLQTQERAA  RLAEFRRANYACVAPELIESMYKLLYLQRLHPGEPQHRIVPNTEVLEVAR  SEDGARVRLTVGSTLSTGGAPAAGEYAAVFLGTGYHRPASSLSFLAGLSQ  EYPTLGEFLSGAAPALAVGRDYALTGSGARVFVLGFGETTHGLSETLLSV  GAVRAGEVVNALHLPAASAASAEVTTPVATKVERGRSHSVQPVHVGTLTP  DSERSPERD |
| *A05283* | MNEKDNGADARNGPGLANTAHGLPNDTLDDSGGGGQPAGRASNVGRQPNG  PAIHGGHKDSVEAYWEKKLQGWTLEPFPDLTGLRTRAKGSERGSATVKVD  EGVLPSALAFQAAWAFLLHMYAEAESPDVVFGVEHGARRLTSEEAQGTNG  AILSKLAADEKESGAFPVTDAVNGLGFDSAIYFHGVADDDDNEKLAVRIA  VDELSAAFSPQVLTQPAAECILRQLAEVAVSIEANPTRLFLDNAAEIRED  LQAALNASPEKLPVPDGELLHSAFESNARERPDKLALWFHAEDGSEVKWT  YGELDKKASVLAKAIQDAAGDAGIVDAAVPLCMEKTPELYVAILGVLKAG  GAWCPIDPAFPALRKQDLLTRAGGPVVLVGTAAERDALVADGALPADRAV  TVLAVADIIAHETDVPVVTCGARPSTLAYLIWTSGTTGAPKGVPIEHSAA  VQAIAALHRDIPYSVDDDVRCIQFSAFTFDVSVQDFFYTWGAPCGILCSA  SRSLLLGQFPDLCNAFEITHAFLTPAFMATTSLAACKTIKTLTSIGEKLP  DAVADQWCAPGVVSVNTYGPAESTIVSTVRTWTPGEGTKAHNVGRPLSTI  SCFAVKDHRVLPRGAAGELAIGGFQNARGYFSQPDKTAAKFVAHPRAGRV  YLTGDVVRILHDGSIEFVGRTDDLVKLGGVRVELSEISALIAPSDRGEWG  PVATLQLGRADRPQKVICAFVAAKALAGEPSIVCEAAANVAAEARERAVA  TLPSYMVPNVFLVVPRIPTTPSNKIDRTALARVYAEVDLAAWEALLARKA  PQPGGKAPENEKEKEAVEAIRAAVAKLTGAPLAVVGVDTPLNALGVDSIR  AIQLAARIRSLGLGRDVAVLDVLEHPTARQLAAFVVSDPAGGDDSEAREI  NEALKAFNSQWLPRIQQQLHDKVGETTQVQMVLPCLPLQEGMLSERALHH  RAYWSHTCLELDPSVDADKLRDAWGALAKEYDMLRTGFVQPTGNYSDDVL  YAAPYVDWTEVKMGDVASLKDLASVRAEQVMKDALTTARPPWALSLLSNA  DGQRMLVLSAHHSIYDADTLRILLEDAFAAYHGRPLPSRPALLSVVASQR  NTESDAGQFWSTALQPFADPEPHGLPDLTGRTRPKDSRAPGHISHRHATT  SSREELEAAGRALGGSLGHLAQVAWAVVLGAYTEAQRVVFGETLSARRDP  RALGPLLTVSPVPLDVGELVTARMAVAALVKLAEESVGRRGVPVSVYREA  LHRGKDKSAWDAMFVLHPETEDEDGAVAALWRVVPNVVPLYVEHGWALNV  ELRKGGVELDIWANSALISPTQLALLTDQTDAVLSLLAKHPDVQFGALLS  QLPLQFLSYSAPTPPPSIVDAPNRHPTWWLERYAREYPDWTAAIVASRIE  EDGADTTSWTYKELDDAANRVAHFILSKGIRGRTVAFCMGRTLPAYAYQL  GIFKTGNCYLPVEEDLPAARKVLLVHDSDAALVFTTTEQAANFQDVRASV  EIITVDQSTHRAELARQPVTVPDVDYDRYAPGYLLYTSGSTGLPKGVLVS  HGNLVSFIECLYELINKHCPVPPFAGRGRFLGRTSIAFDVHLLEIFAAFR  FGMATASAPRHIILADLGNTLRHLKITHSCLVPSLLERSGLVPADVPDLR  WASVGGEKISARIIEVWGNDPGGIVLFNAYGPTEVTIGCSMARVTGTSSA  RDIGRIFDGNQAHVFRPGTQQLTLRGQPGELCVSGDLVGIGYLNRPDAKG  FVTTENGTVLYRTGDMVRLLADETIEYLGRSDDQTKIRGQRLELAEVSEC  LRAASDENLAVASLVAKHPSLSRDLLLAFFSRVTRAQRDATDVPAVLHTE  AGLSERLLTACRDRLPAFMVPDLIIAVDFMPVALISRKVDSKLLRKVFSD  APLDALMGDGTQPTRALTDDEKRVQDVILSIVPFISEPITNRTTTLQLGL  DSIGAIRLAARLDAAGLTVPVAFLVRGPSIESIAGKATKEATDQHAIPLS  RLEDLQAFARRARAVLGPSRDIQDIFPALPLQESLVARSLDSDVPLYVNH  VLLDVQPDRVEALLHHLSDTITANAILRTCFIPVDRKVAQVVLSCGSYPD  VVTCHAVEGESLVLLLREIELRVSNQFAIQRTPFSVIVNYVNTRSLEDAR  RFYETYLKNLSPQPVLPSVNVVDKVQGDSEHVLTHQHTPLQLIQQWAGHY  DALFNALFTFTRSDGLRLDPQLWRELPGAFGIDIAQYDEESWDEVESRIR  ACVAEICDVQLEAVTKNVAFLRLGLDSISTIRMAQRLRSVGVDVRSADIL  RFPCIGALAEHIAATKAGAERRDDPVLLFSKAGEKLWQEHVESIPTLTEK  DRIQQVVPATALQASMLSSTLASEGRLYVISHPLKLHHTVDIPRLRAAWE  RLVNHVDILRTSFHQAVDLPWLVMHHALYDGTSLEYLLQDLERAYEGQPL  VERPQFSLALPYLLHRPQDEAEFWQRRLAGYAPVQVRSSAPSAQAALYEC  LSTLSTSRIDAACKTTEVTVQAVALLAWAKVYASLTGSSDVVFGHVISGR  SIPLEGALSVAGPLFNTVPARVTFSSDSDTNADAAKTLHIAHALAEPYQH  TPLRVIQNAWRGASNAALFDALFVFQRTEQLLERSTAPPWTPFQSENEPY  EPEYALNAEVEYAEAAIEDNGAFSPDEQALREAVCAVARVPLERLRPRTP  FYTVGVDSISVLQIVARVRRGFAIGDLLTGMHVRGAIAARDARLRKTEGG  GEIVPTDVQATVLAQLGLEAKQVEYVLPVLPGQRHHVAAWLATGRTFYEL  PFAYRAPKPLDREQLSTAWARLRARHAVLRTVFAATSSPELYQVVIHDST  ADTTYAYIEGDEDLEALVKTKMTEAMSAPSNMRSPPVRAFHVRGKDGHDA  FVLVIHHALYDGWSLPLLIADLCTLYGDASAALEPAPSFPDLVKHIVART  DSTSQAYWAEYMRGARPTRLTATPGASRDAFLFAQNVLRADGLTPALLLR  ALARALPLEDAEAVVGLFQAGRSTPFADIERVAGPTLALLPLRIGGAARD  LEADLATRAPHEQYDGAEEWDGLFNVYVNVLWHADRLWDDDAKMGGVLER  MDGGEQVGDATDYAPHAPIAGRTAVDLLDVSKVPKESVSIDLALNPRTGG  VDVGVRCEGVLLGVSDIQQLVDAFVRNVRAMT |
| *A01433* | MSETGSFKGGADDQLRKRASRTEVSVHVVDTQQGVLKAEATRRVWGPRSK  IFLYLGIALVAYVYSLDGTTTYLYQAQATSAFDQHSTLAAIGVATAIILA  VVKPIAAKLSDVLGRAEAYLMAVIFYTIGYIVVASCKKISTYAGGSERFD  TLIMQRYTKPSLSPHLHLRQCSDTDFAADMTTLRWRGLVSSFVSAPFFIN  AFVSANIQAAVMAGPGWRWGYGMFAIIIPICTFPIISTLTWGQLRAKKLG  VLATTYHGEQQVVFASRLSVKQRIINAMIDIDLVGLILFAAGWALVLVPL  TIVNRGTQKWSSPNIIAMLTLGPIVLILMVLYEAKYSPKPVFPLRFFKNA  TVLACALIGLFDFISFYLQYSFIFITHSDWSLKNQGYFAYTQTLSLTFFA  IMAGAIQFSTRRLRWLLLAGVVIRLAAVGAMIKTKGAHGSTFGLVFTQVM  QGAGGGIAALSTQVAAQGSVPHQDMAAVTAMILLFSEIGNAIGNAIAGAI  WKNLMPGQLEQRLAGHLNQTEIDTIFGSITNAVTYADRPEVFNGIVGAYD  FVMTRLLIAATVVAVAPVICAWFVHDIHLGDKQNAVEADDDSEVDSPTNM  KS |
| *A10927* | MSARNSRDLSEKPAGEPPAAPPAAADDAVSHYDEKHHGTDVQVHETGAGA  DIEHMKSQPPGVTRIEAFSRAFGHSRWAMYVLYGAVAGVMVATCLDGSTV  YTYEAIAASSYGEHGELLAAIATVTSIINAVSKPFIAKICDLSSRQTSIL  VMLCFYVLGYVVVAASHTADGFAAGRVISVLGNSGLQFSVGIIISDLSPL  QWRGFVEGLSAVPWIPFSFVGPNISGPLIERGLWRWGYGMFCIMWGTQYS  VVWAFIIAMMTLGAIFVLGFWLWELRFASQPLMTKRIFFNRTFQLAMGID  FCYFIGGYMQLVYYSSYVYLVKDWTATEWGYFNNELTVALCGGGLIVGVL  MRMIHRYKIVQLVGLMLRCIGVGIIYYARGENASTAALVMGTALAGLGGS  CSVIGTQVATQASVPHHDLASVIALLSLFTTIGGSIGSAIAGAVWTNTMP  GNLLKEGIPADQVPAIYGGLLGIHDQFPLGDPLRQAVIRAAGKTLEPLFV  ASLVITFIGVFFGSMMPNFHLGKSHNAIDGTDVAGRKIAESDRAVLPPKP  ENETAWQWIKREFF |
| *A00549* | MAAPQAVNEKAPPVDDDASIRKDPEIGSDLADSRADAKRASLQPGVGKIE  AFARVFKQHRWVMYVLYVAVIGVMLASCLDGATTSSYEPIASSDLGQHGS  LLSTIATVTGIMNAVSKPFIAKIADISSRQTAYLVVLVFYVVGYAVMAGA  SSPAAYAAGQCVSTIGDSGMSFVTGIIVADLSPLEWRGFIGSLGSAPWLW  FAFIGPNISGPLIDRGQWRWGYGMFCIITPVMLIPAILILFAADRRAKAI  GEVNIAESLYARRYKTDHNGAEVPEAFIIAMMTLGAVLLIVFAVWDVKFA  ASPVMTKRIFFNRTFLLAMAIDFCYFAGGYLVSVYHSSYVYVLKPWSNTK  WGYFNNVMTMGLCGGGLIVGLLMRATHRYKFIQLSGLCIRAIGVGLVNYA  VGDNASDVALIFSRLLGGIGGSCSVVGTRVASEASVPHHDLASVIALLSL  WTNLGGSIGSAIAGAVWTNNMPKYLVQEGIPHDLASTLYRRLAAVHSKYE  LDDPIRQAVIRAATRTLKPLFLSGLLVAILSIGFGVFMPNYRLGKTHNMV  MQPPVSR |
| *A16413* | MEYLLKRSSNKTPGLPPPSAADLAYRHKADLLRPKWIFYAIVAYIALAMV  VRFTFAFGAWLRLRRLRGATAAAVVNSQEYGSPAQQRVSLRRLPAALTTS  FRTVAYRWKVPLGDWHHMNGLTVFTAIAYMTVLFTLSFSSAHHNDPNLWA  NRVSHVTSLQWTFVVALSGKNNIISVLTGIGYEKLNIMHRIAARLIGPDS  MKEVHHLLGLVSGIAYLFVFFLSLRPVRKWAYDFFLPVHIVLVLLTIISG  YYHSFNFKYYMWSTFVVWGFDRLCRFLHLIYNNRLWRLLILRKSSAMTAQ  AELLRDDAIRLTLRRNMSWSPGHHVYVTIPAVSHWPLEAHPFSIATHALE  GQTEDPDGRRVVLIVRPREGLTRRLANRLEHETEGPEELTLLVDGPYGSP  PSIISYPTVVLIAGGTGASYTMSYLLDIIHNFKNKNCATRRVVFAWAVKA  RTNLDWYSGTLESIAADVPEGLSLDVHVYVTQQDDSKSEGSQDGHEKSSS  VVPFRLSHGRPDIAKIISDEVTSTNDVVSVGVEMAMTARRAMQNFGFSSV  LKGGATINAHFEMFTNT |
| *A12570* | MPMALLPFLLLAAYAAPGLGALVEKWDITFVNANPDGLFERRVVGVNGSW  PPPPIELTVNDTLRVHATNKLDKPTTLHHHGMFFNGTSYFDGAAGVSQCG  IPPGQTFTYDVDVANSGQWGTENATLPFEPGKTYRLRIVNTSAFSMFFFW  IDGHDMRVIEVDGTDVQEFPASLLSLTVAQRYSVLVTARNDTSSNFLIHA  NMDTDMFDVVPDGLNPNTTATVVYDTNAATADGVDKDAYFDFPDISLVPV  IQEPQQRATHQVYRKICMIYACPNVLQIVLNVLFDTMTDGTNRAMFNNIT  YNTPVVPSIFSELSLGQDAVQAELYGPTSFVLNQFDVVEIKVVNFDAGKH  PFHLHGHKFQIVHKSTDFTSDDPTLNPPLIEGQINPVRRDTVQVPSMGSA  SLRFVADNPGAWFFHCHIEWHLEAGLAVTFFEAPLEAQQKLKPPQFMFDQ  CKAMGVPTAGNAAGHTGADLSGLKVGPFQQKLGWRPKGIVAMTGCVLTAT  LGMLTVAWYAWGGHISDEEVEDEVRRRQEVRSARPSIFSRMKKN |
| *A12568* | MAKDLFSVPIFFIVFRETLEAAIILSVLLGIVDQLIYKNAVAAPGTTTTT  SDGDVEALPPGWEKRLVRKLRIQIFAGALAGFLIALAIGAAFIAVWFTQA  SDLFGKAEELWEGVFDLVASIIIFVMGVTMLRMDRAKAKWRVKLMHVFDG  KKLDRTSRNSRWALFLLPFITVLREGIEAVVFVGGVSLGQKAESIPIAAI  VGIICGVVVGYLIYEFASRTTLTVFLVVITNFLLLIGAGLFSKAVGSFEA  DKFNRLTGGDAAEAGDGPGSFDVRGSVWHLNCCNPENKFDGSGWTIFAAI  FGWTNNGTLGTILSYVFYWIAVMVALVYMKFTEGRTTLFGRESKVATARR  LAHQRPFSASHRAQDGKPPQELGVPFSELTVGIPKETFPGERRVAITPQN  VTLLLKKGFKRVLVEDGAGNEAQFPNAQLKAAGATIVDKATVLGDSDILL  KVRPPLLPDQNSDEIDKLKKGSTLISFVYPAQNPQLLDVLKQKELTLFGM  DCVPRISRAQVFDALSSMANIAGYKAVLEASNNFGRFLTGQVTAAGKIPP  TKVLIIGAGVAGLSAIATARRMGAIVRAFDTRPAAREQVESLGAEFLEVA  VKEDGTGTGGYAKTMSAEFIAAEMALFMEQAKDVDIIITTALIPGKPAPK  LITKEMVAAMRQGSVIVDMAAEAGGNCEVTKPGQQYVHDGVTIIGYADLP  SRLPTQSSTLYSNNIVKFLLSMTPAGVNNRFHLDLEDEVIRRSIVLHKGS  LLWPAPLPPPTPPPQISEAKKEEAKEVVALTPWKKAVRDVAVVTGGMTSL  VALGKATTPAFMANFFTFGLAGLIGYRVVWGVAPALHSPLMSVTNAISGL  VAVGGIFVMGGGYFPHTFPQWLAALSVLLANVNIFGGSVISGRMLDMFKR  PTDPPEYTYLYGVPAAAFTGALLMSASTGAAGLVQAGYLASSMLCIGSLS  GLGSQTTARQGNMLGMLGIGSGVLTSLVAVGFPPDILAQFGALTALGALA  GTWIGRRVTATELPQMVAALHSVVGLAAVMTSAASVLAATGDHLTTLHLV  TAYLGVLVGGITFTGSIVAFLKLAAKMSSKPLILPGRHAINAGLLAANAA  TMGAFVTMAPTAPVVGALCLLGNTALSFIKGYTLTAAIGGADMPVVITVL  NAYSGFALVAEGLMLQNDLLTSVGALIGCRAMNRSLPNVLFGGIGTPTAA  VEGPKIEGTITKTNIEEAVDILYNAESVIITPGYGMAVAKAQYAIAEMTR  LLRSKGINVRFGIHPVAGRMPGQCNVLLAEAGVPYDIVLEMDEINEDFGE  TDVTLVIGANDTVNPIALEPGSAIAGMPVLEAWKSKNVIVMKRGMSSGYA  DVPNPMFFMPNTKMLFGDAKDTCDALKKGIEEKTKE |
| *A17439* | MSHLQYFSYAGVGERKRQQMHYNQAVRIGDRIECSGQGGWDVDTGIVHSD  LLTQIAQAYANVDLALRTAGGAGWSQVYRVRIYYTRAAAGEEAFAAMIDG  LKRWCPAHQPILTGVEVAGLADPGMEVEIEVAAHVPSGHY |
