# Supplementary material for: Transcriptome sequencing reveals iron acquisition–related genes and iron acquisition systems in Auricularia cornea
Source: BMC Genomics. 2026 Feb 26;27:336. doi: 10.1186/s12864-026-12654-6 (PMC13041173; doi:10.1186/s12864-026-12654-6)
Supplement: Supplementary file 3 — Supplementary Material 3. [file 12864_2026_12654_MOESM3_ESM.docx]

Table S3. Gene ID and functional annotation related to iron acquisition in *A. cornea*

| Gene Function | Gene ID | Databases | Functional Annotation |
| --- | --- | --- | --- |
| L-ornithine N5-monooxygenase | *A05285* | KEGG | K10531；pvdA, SIDA; L-ornithine N5-monooxygenase [EC:1.14.13.195 1.14.13.196] |
|  |  | GO | GO:0019539; hydroxamate-containing siderophore biosynthetic process |
| Nonribosomal peptide synthase | *A05208、A05283、A04921* | KEGG | K22148; SIDC; ferricrocin synthase; (RefSeq) nonribosomal peptide synthase 2 |
|  |  | GO | GO:0019539；hydroxamate-containing siderophore biosynthetic process |
| Siderophore-iron transporter | *A00549、A01433、A06780、A08226、A10927、A12157、A13200、A13374* | KEGG | K08197; ARN; MFS transporter, SIT family, siderophore-iron: H+ symporter |
| Ferric reductase | *A06017、A06018、A06183、A06622、A12095、A14664、A16413* | KEGG | K00521; ferric-chelate reductase [EC:1.16.1.7] |
|  | *A03586* | Pfam | PF08030:Ferric reductase NAD binding domain |
| Multicopper oxidase | *A06630、A09885、A12570* | KEGG | K19791; FET3_5; iron transport multicopper oxidase |
| Iron Permease | *A06503、A12568* | Pfam | PF03239:Iron permease FTR1 family |
| Ferrous ion transporter | *A04820、A12437、A14566、A17439、A17443、A17450* | KEGG | K12346; SMF; metal iron transporter |
