# Supplementary material for: Transcriptome sequencing reveals iron acquisition–related genes and iron acquisition systems in Auricularia cornea
Source: BMC Genomics. 2026 Feb 26;27:336. doi: 10.1186/s12864-026-12654-6 (PMC13041173; doi:10.1186/s12864-026-12654-6)
Supplement: Supplementary file 4 — Supplementary Material 4. [file 12864_2026_12654_MOESM4_ESM.docx]

Table S4. Log2(FoldChange) of key iron acquisition genes across treatments

| Gene Function | Gene ID | Log2(FoldChange) | | | | | | |
| --- | --- | --- | --- | --- | --- | --- | --- | --- |
|  |  | JST-CK vs JST-T | YJ-CK vs YJ-T | ZST-CK vs ZST-T | JST-CK vs YJ-CK | YJ-CK vs ZST-CK | JST-T vs YJ-T | YJ-T vs ZST-T |
| L-ornithine N5-monooxygenase | *A05285* | -4.217 | Nodiff | Nodiff | -1.729 | -1.455 | 1.5918 | Nodiff |
| Nonribosomal peptide synthase | *A05283* | -1.454 | Nodiff | Nodiff | -2.439 | Nodiff | Nodiff | Nodiff |
| Siderophore-iron transporter | *A01433*  *A10927*  *A00549* | -2.499  -1.878  -2.102 | Nodiff  Nodiff  Nodiff | Nodiff  Nodiff  Nodiff | Nodiff  -1.392  -2.314 | 1.5914  Nodiff  Nodiff | 1.6034  Nodiff  Nodiff | 1.5304  Nodiff  Nodiff |
| Ferric reductase | *A16413* | -3.604 | Nodiff | Nodiff | -7.435 | Nodiff | -3.723 | Nodiff |
| Multicopper oxidase | *A12570* | -4.193 | Nodiff | Nodiff | -3.512 | 1.3735 | Nodiff | 2.3401 |
| Iron Permease | *A12568* | -1.299 | Nodiff | Nodiff | -1.550 | Nodiff | Nodiff | Nodiff |
| Ferrous ion transporter | *A17439* | 1.289 | Nodiff | Nodiff | -6.625 | Nodiff | -7.996 | Nodiff |
