# Supplementary material for: Transcriptome sequencing reveals iron acquisition–related genes and iron acquisition systems in Auricularia cornea
Source: BMC Genomics. 2026 Feb 26;27:336. doi: 10.1186/s12864-026-12654-6 (PMC13041173; doi:10.1186/s12864-026-12654-6)
Supplement: Supplementary file 5 — Supplementary Material 5. [file 12864_2026_12654_MOESM5_ESM.docx]

Table S5.Siderophore production capacity of the tested strains

| Strains | strain type | Mycelial growth matrix | Siderophore production |
| --- | --- | --- | --- |
| ACW1 | Cultivated strain | PDA medium | Positive |
| ACW8 | Wild strain | PDA medium | Positive |
| ACP4 | Wild strain | PDA medium | Positive |
| ACP16 | Cultivated strain | PDA medium | Positive |
| ACP36 | Wild strain | PDA medium | Positive |
| ACP126 | Cultivated strain | PDA medium | Positive |
| ACW8 | Wild strain | Cultivation medium | Positive |
| ACP16 | Cultivated strain | Cultivation medium | Positive |
| ACP36 | Wild strain | Cultivation medium | Positive |
| ACP126 | Cultivated strain | Cultivation medium | Positive |
