# Supplementary material for: Transcriptome sequencing reveals iron acquisition–related genes and iron acquisition systems in Auricularia cornea
Source: BMC Genomics. 2026 Feb 26;27:336. doi: 10.1186/s12864-026-12654-6 (PMC13041173; doi:10.1186/s12864-026-12654-6)
Supplement: Supplementary file 7 — Supplementary Material 7. [file 12864_2026_12654_MOESM7_ESM.docx]

**Additional Fig S1.png** **Title of data:** Sample relationship analysis. **Description of data:** (A) Heat diagram of the Correlation analysis of three biological experiments for each sample of the CK group and the T group. (B) The top 3 principal components (X-axis, PC1; Y-axis, PC2; Z-axis, PC3) deciphered 67% of all. Note: JST, YJ, and ZST represent the samples of mycelia, primordia, and fruiting bodies, T represents the treatment group, CK represents the control group, and the numbers represent the replicates from 1 to 3, respectively.
