# Supplementary material for: Transcriptome sequencing reveals iron acquisition–related genes and iron acquisition systems in Auricularia cornea
Source: BMC Genomics. 2026 Feb 26;27:336. doi: 10.1186/s12864-026-12654-6 (PMC13041173; doi:10.1186/s12864-026-12654-6)
Supplement: Supplementary file 8 — Supplementary Material 8. [file 12864_2026_12654_MOESM8_ESM.docx]

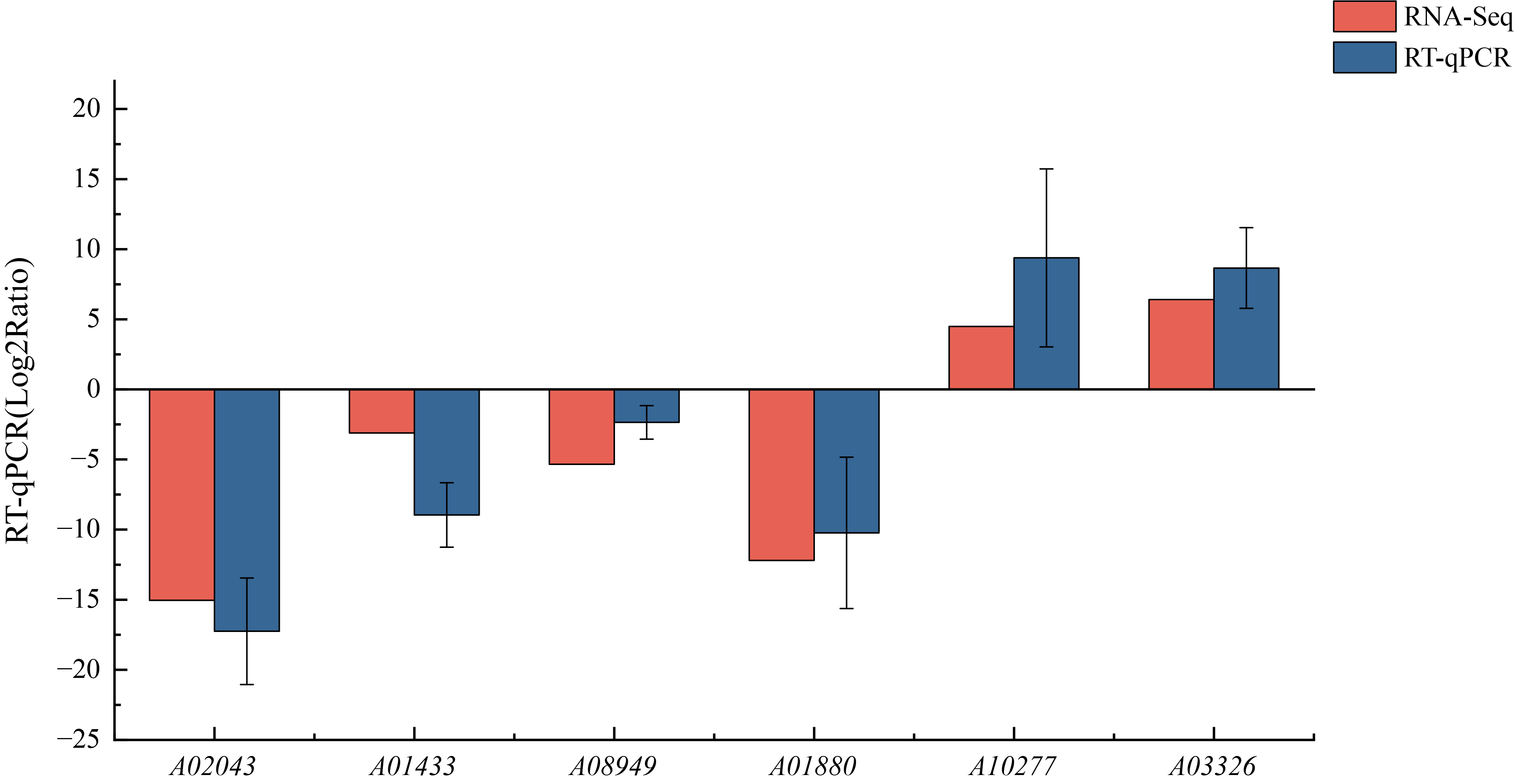


**Additional Fig S2.png** **Title of data:** Validation of the RNA-Seq of 6 selected DEGs in the *A. cornea* transcriptome by RT-qPCR. **Description of data:** The blue column represents the results by RT-qPCR, and the red column represents the results by RNA-Seq. The error bars represented the standard deviation of three biological replicates and three replicates of RT-qPCR runs.
