# Supplementary material for: Transcriptome sequencing reveals iron acquisition–related genes and iron acquisition systems in Auricularia cornea
Source: BMC Genomics. 2026 Feb 26;27:336. doi: 10.1186/s12864-026-12654-6 (PMC13041173; doi:10.1186/s12864-026-12654-6)
Supplement: Supplementary file 9 — Supplementary Material 9. [file 12864_2026_12654_MOESM9_ESM.docx]

**Additional Fig S3.png Title of data:** GO enrichment analysis of DEGs between the T group and CK group. **Description of data:** The x-axis presents the number of DEGs. The y-axis represents the biological processes, cellular components, and molecular functions of DEGs.
