# Supplementary material for: Transcriptome sequencing reveals iron acquisition–related genes and iron acquisition systems in Auricularia cornea
Source: BMC Genomics. 2026 Feb 26;27:336. doi: 10.1186/s12864-026-12654-6 (PMC13041173; doi:10.1186/s12864-026-12654-6)
Supplement: Supplementary file 10 — Supplementary Material 10. [file 12864_2026_12654_MOESM10_ESM.docx]

**Additional Fig S4.png** **Title of data:** KEGG pathway enrichment analysis of all DEGs in the mycelium period between the T group and the CK group. **Description of data:** Red boxes or lines represent upregulated genes, and blue boxes or lines represent downregulated genes. (A) Nitrogen metabolism (map00910); (B) Fatty acid degradation (map00071); (C) Other glycan degradation (map00511).
