# Supplementary material for: Transcriptome sequencing reveals iron acquisition–related genes and iron acquisition systems in Auricularia cornea
Source: BMC Genomics. 2026 Feb 26;27:336. doi: 10.1186/s12864-026-12654-6 (PMC13041173; doi:10.1186/s12864-026-12654-6)
Supplement: Supplementary file 11 — Supplementary Material 11. [file 12864_2026_12654_MOESM11_ESM.docx]

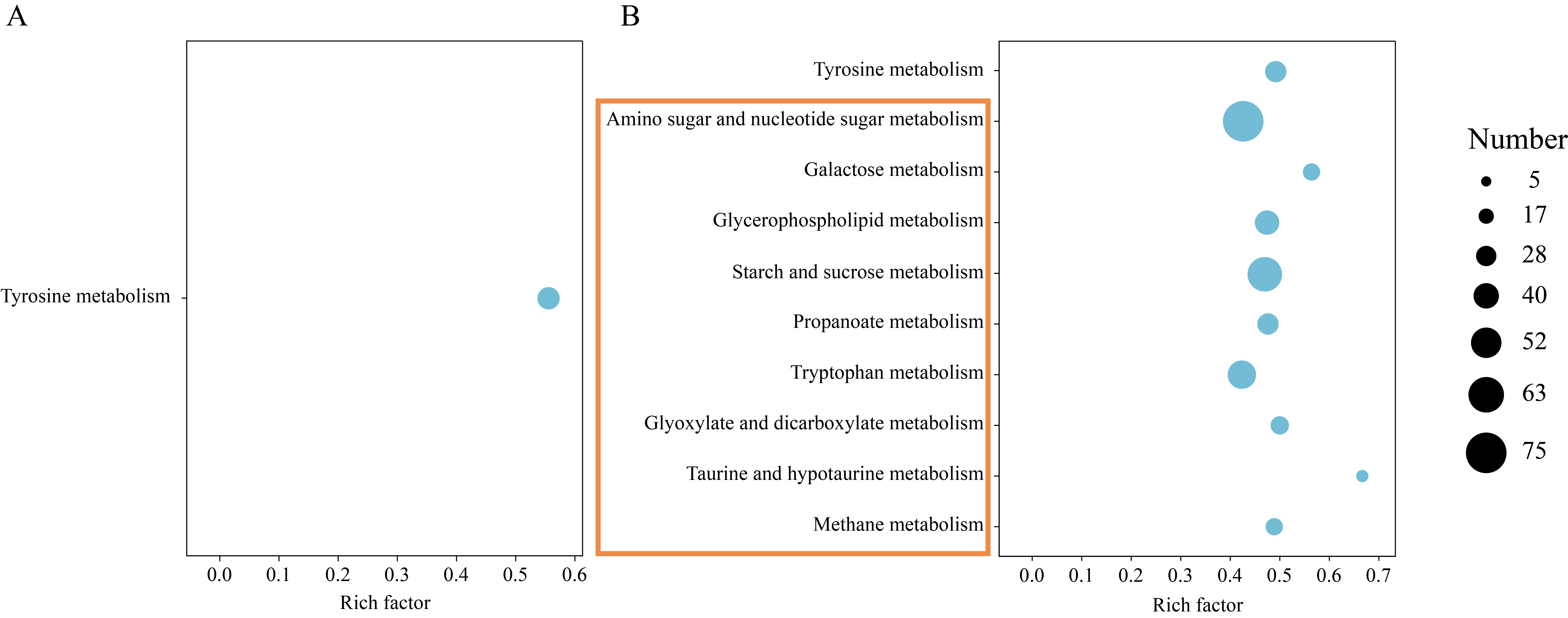


**Additional Fig S5.png** **Title of data:** Scatter plot of KEGG pathway enrichment analysis of all DEGs between the mycelia period and the primordia period. **Description of data:** (A) CK group, (B) T group. The Y-axis represents different KEGG categories. The X-axis represents the richness factor. The size of the dot indicates the number of DEGs involved in the pathway. Orange boxes represent KEGG pathways that exist only in the T group.
