# Supplementary material for: Transcriptome sequencing reveals iron acquisition–related genes and iron acquisition systems in Auricularia cornea
Source: BMC Genomics. 2026 Feb 26;27:336. doi: 10.1186/s12864-026-12654-6 (PMC13041173; doi:10.1186/s12864-026-12654-6)
Supplement: Supplementary file 13 — Supplementary Material 13. [file 12864_2026_12654_MOESM13_ESM.docx]

**Additional Fig S7.png** **Title of data:** Phylogenetic tree of key iron acquisition proteins. **Description of data:** (A) Phylogenetic tree of L-ornithine N5-monooxygenase(A05285). (B) Phylogenetic tree of nonribosomal peptide synthase (A05283). (C) Phylogenetic tree of siderophore–iron transporter (A01433). (D) Phylogenetic tree of siderophore–iron transporter (A10927). (E) Phylogenetic tree of siderophore–iron transporter (A00549). (F) Phylogenetic tree of ferric reductase (A16413). (G) Phylogenetic tree of multicopper oxidase (A12570). (H) Phylogenetic tree of iron permease (A12568). (I) Phylogenetic tree of ferrous ion transporter (A17439). Node values represent bootstrap support based on 1,000 replicates.
