# Supplementary material for: Transcriptome sequencing reveals iron acquisition–related genes and iron acquisition systems in Auricularia cornea
Source: BMC Genomics. 2026 Feb 26;27:336. doi: 10.1186/s12864-026-12654-6 (PMC13041173; doi:10.1186/s12864-026-12654-6)
Supplement: Supplementary file 14 — Supplementary Material 14. [file 12864_2026_12654_MOESM14_ESM.docx]

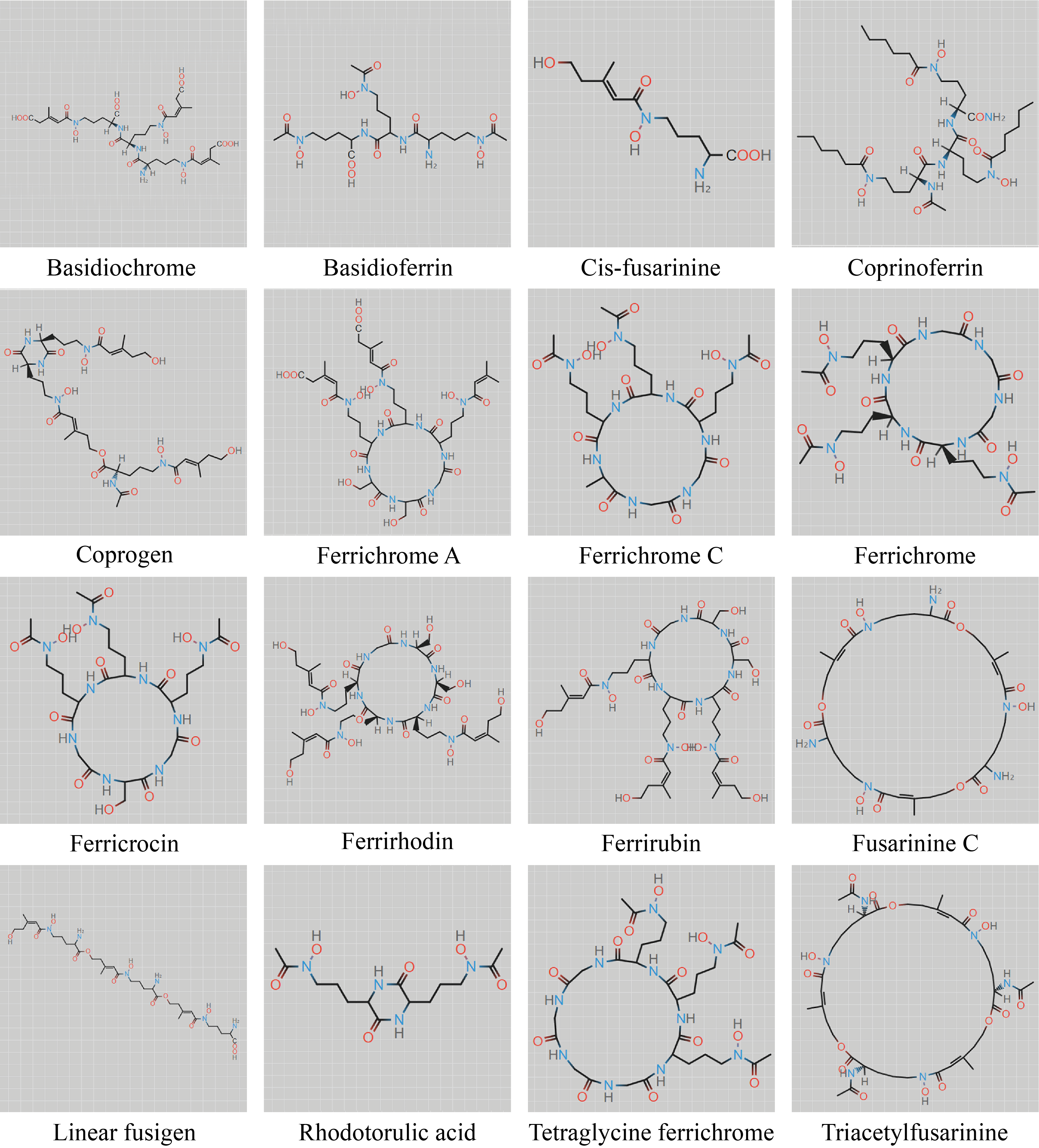


**Additional Fig S8.png Title of data:** Molecular structures and names of 16 structurally unique siderophores.
